# Cutaneous protothecosis in kidney transplant recipient^☆^^☆☆^

**DOI:** 10.1016/j.abd.2019.08.022

**Published:** 2020-01-21

**Authors:** Valeria Romero Godofredo, Milvia Maria Simões e Silva Enokihara, Jane Tomimori, Marilia Marufuji Ogawa

**Affiliations:** a Department of Dermatology, Universidade Federal de São Paulo, São Paulo, SP, Brazil; b Department of Pathology, Universidade Federal de São Paulo, São Paulo, SP, Brazil

**Keywords:** Harmful algal proliferation, Opportunistic infections, *Skin diseases, infectious*

## Abstract

Protothecosis is a rare condition caused by the aclorophylated algae of the genus *Prototheca*. In humans, protothecosis, caused mainly by *P. wickerhamii*, manifests itself in three forms: cutaneous, articular and systemic. It can occur in both immunocompetent and immunosuppressed individuals, being much more common in the latter. We present a new case of protothecosis in Brazil in a kidney transplant recipient.

## Introduction

Protothecosis is an uncommon infection caused by the aclorophyllated algae of the genus *Prototheca*. Five species are known, two of which affect humans: *P. zopfii* and *P. wickerhamii*, the latter being the most common.

The first case of human infection was described in 1964 as a cutaneous ulcer located on the foot of a rice grower in Sierra Leone.^1^ A little more than 200 cases have been reported worldwide, with 10 in Brazil.^2^, ^3^, ^4^ We report a new case of protothetosis that occurred in a renal transplant recipient (RTR) in Brazil.

## Case report

**Figure 1:**
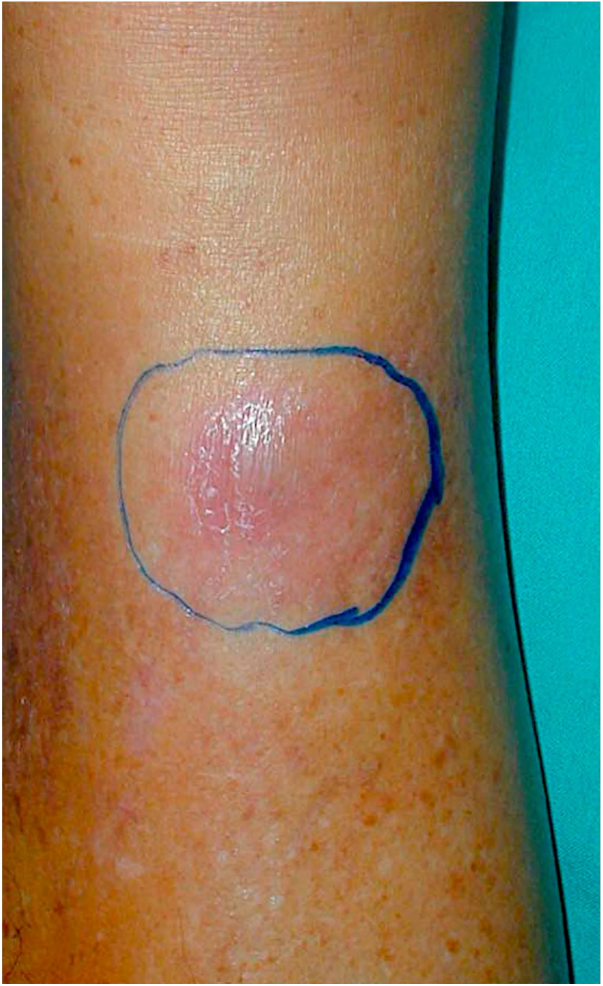
Erythematous nodule in the distal third of the right leg.

A 60-year-old male patient, a skin phototype IV, born in Caetité-BA and moved to São Paulo-SP 35 years ago, reported a nodular lesion 6 months ago on the right leg (Fig. 1), with no history of previous trauma. Regarding his personal history, the patient was RTR 15 years ago, due to arterial hypertension, and had been treated for multiple previous squamous cell carcinomas. He had already used cyclosporin and azathioprine. At the time of the infection, the patient had been using prednisone 5 mg/day and sirolimus 1 mg/day for 5 years.

**Figure 2:**
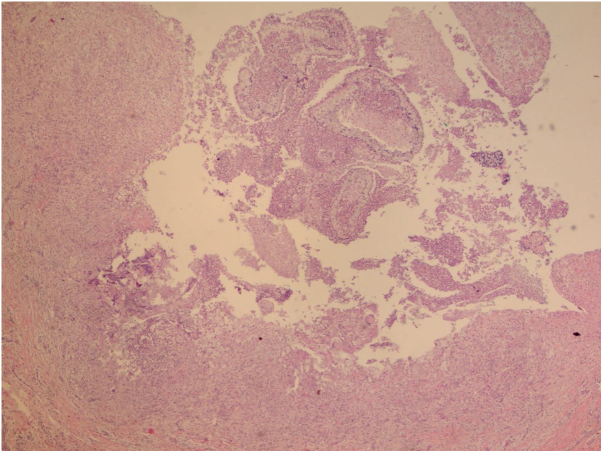
Presence of a granulomatous inflammatory process with suppuration. In the middle, the sporangia (Hematoxylin & eosin, x40).

**Figure 3:**
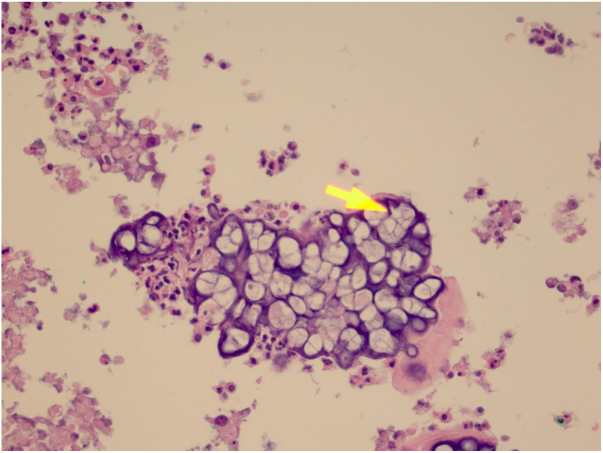
Presence of sporangia grouped within the cytoplasm of macrophages (Hematoxylin & eosin, x400).

**Figure 4:**
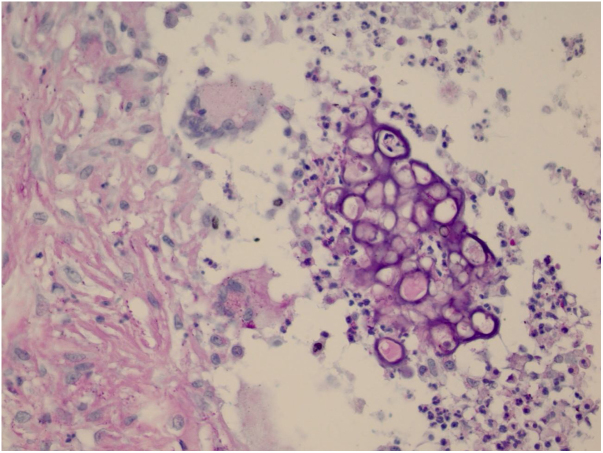
The sporangia appear stained by PAS (x400).

**Figure 5:**
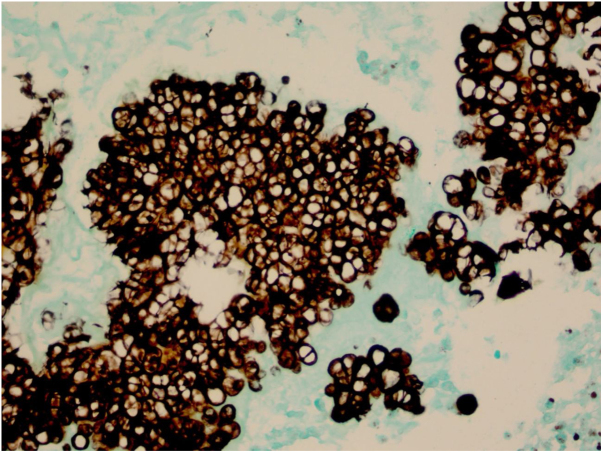
The sporangia appear stained in black by Grocott (x400).

The initial diagnostic hypothesis was subcutaneous mycosis and the patient was referred for complete lesion excision. The material was sent for histopathological analysis and granulomatous inflammatory process was observed with suppuration and sporangia grouped within the cytoplasm of the macrophages (Figure 2, Figure 3). The sporangia were stained by Period Acid-Schiff (PAS) and Grocott, and presented a morula appearance (Figure 4, Figure 5). Part of the skin fragment was sent for mycological examination and fungal culture, with no growth of agents.

In the postoperative period, the surgical wound was allowed to heal by secondary intention and was initially performed with compressive cotton dressing, then dressed daily with topical mupirocin for 3 weeks, and silver nitrate 10% until complete healing. The patient was treated with fluconazole 150 mg/day for a period of 3 months, until complete healing of the lesion.

## Discussion

The algae of the genus *Prototheca* do not present chloroplasts, organelles that contain the chlorophyll pigment, so they do not have the capacity to perform photosynthesis. They need a heterotrophic source of nutrients, such as organic carbon and nitrogen, and the skin is the most frequently affected organ.^5^

*Prototheca spp.* is widely distributed throughout the world. Algae are found in soil, in the decomposition of plants, in water, and in certain food items. They also exist as a saprophyte organism in the human skin, nails and respiratory and digestive tracts.^1^, ^6^

*Prototheca spp*. resists chlorine treatment, sewage treatment and intestinal digestion. This contributes to its persistence in sewage, dissemination by domestic animals and permanence in the environment. It is also resistant to milk pasteurization, representing a problem for the consumption of dairy products.^6^

Human protothecosis is rare, but the incidence is higher in immunocompromised patients (local or systemic corticosteroids, immunobiologicals,^7^ hematologic malignancies or cancer, diabetes mellitus, AIDS, solid organ and bone marrow transplant recipients, alcoholism or autoimmune disease). In the literature, only 219 cases of human protothecosis have been described worldwide, of which 11 were reported in Brazil,^1^, ^4^ including this one. Among the cases of human protothecosis, there are only 15 reports of cases in solid organ receptors,^4^, ^7^, ^8^ 9 in renal transplant recipients and 1 in liver/kidney transplantation. In Brazil, it is the first case described in a renal transplant recipient.

The pathogenesis of protothecosis is unknown. In most cases, the source of the infection is exogenous contact with contaminated soil or water, commonly occurring after traumatic inoculation. These infections were also described as complications after surgery.

In man, protothecosis is caused mainly by *P. wickerhamii* and manifests itself in three forms: cutaneous, articular and systemic, with an acute or chronic course. The cutaneous form is the most observed. Lesions have a slow evolution and variable appearance: plaques, papules, nodules, ulcerations and eczematous eruptions. The most common presentations are vesiculobullous lesion and ulceration.^9^ In the present case, the patient presented a nodular lesion, reminiscent of the phaeohyphomycosis, a mycosis by dematiaceous fungi described in the recipients of solid organ transplantation.

In histopathological examination, there is a granulomatous inflammatory infiltrate, consisting of lymphocytes, macrophages, giant cells and neutrophils. In staining with PAS or Grocott, we can observe sporangia internally containing sporangiospores, which may be inside macrophages or free in the exudate. Sporangia are surrounded by a capsule that can vary from 7 to 30 μm; *P. zopfii* tends to be larger (7–30 μm) than *P. wickerhamii* (3–15 μm).^6^ A feature of *P. wickerhamii* is to exhibit sporangiospores with a rounded central endospore, and this characteristic has been described as morula-like, daisy-like or raspberry-like.^6^ In the histopathological examination of the our patient, sporangiospores were better evidenced with PAS and Grocott staining, so routine screening with special staining for nodular or nodule-cystic lesions in transplant recipients should be included.

There is currently no definite treatment for *Prototheca spp*. due to the rarity of the disease. Amphotericin B appears to be effective in cases of disseminated infection.^5^, ^8^ Azole antifungals are also used, although their efficacy is variable.^5^, ^10^ Our patient had a good therapeutic response with surgical treatment and oral fluconazole, evolving with adequate healing and there was no relapse after 4 years of follow-up.

In conclusion, nodular or nodule-cystic lesions in transplant recipients should be biopsied to screen for infectious agents using special stains (PAS, Grocott, Fite-Faraco). Culturing for fungi and mycobacteria is also recommended. The lesion of protothecosis should be completely excised and healing left by secondary intention. Complementing surgical treatment with azole derivatives is highly recommended.

## Financial support

None declared.

## Authors’ contributions

Valeria Romero Godofredo: Statistical analysis; approval of the final version of the manuscript; conception and planning of the study; elaboration and writing of the manuscript; obtaining, analysis, and interpretation of the data; effective participation in research orientation; intellectual participation in the propaedeutic and/or therapeutic conduct of the studied cases; critical review of the literature; critical review of the manuscript.

Milvia Maria Simões e Silva Enokihara: Intellectual participation in the propaedeutic and/or therapeutic conduct of the studied cases; critical review of the manuscript.

Jane Tomimori: Obtaining, analysis, and interpretation of the data; intellectual participation in the propaedeutic and/or therapeutic conduct of the studied cases; critical review of the literature; critical review of the manuscript.

Marilia Marufuji Ogawa: Approval of the final version of the manuscript; conception and planning of the study; effective participation in research orientation; intellectual participation in the propaedeutic and/or therapeutic conduct of the studied cases; critical review of the literature; critical review of the manuscript.

## Conflicts of interest

None declared.
